# Boosting anti-tumor immunity with TILT-517 oncolytic adenovirus and checkpoint blockade in renal cell carcinoma

**DOI:** 10.1016/j.omton.2025.200979

**Published:** 2025-04-03

**Authors:** Victor Arias, Tatiana V. Kudling, James H.A. Clubb, Elise Jirovec, Santeri A. Pakola, Mirte Van der Heijden, Saru Basnet, Dafne C.A. Quixabeira, Lyna Haybout, Nea Ojala, Susanna Grönberg-Vähä-Koskela, Anna Kanerva, Antti Rannikko, João M. Santos, Victor Cervera-Carrascon, Otto Hemminki, Akseli Hemminki

**Affiliations:** 1 Cancer Gene Therapy Group, Translational Immunology Research Program, Faculty of Medicine, University of Helsinki, Helsinki, Finland; 2 TILT Biotherapeutics Ltd, Helsinki, Finland; 3 Comprehensive Cancer Center, Helsinki University Hospital (HUS), Helsinki, Finland; 4 Department of Obstetrics and Gynecology, Helsinki University Central Hospital (HUS), Helsinki, Finland; 5 Department of Urology, Helsinki University Hospital (HUS), Helsinki, Finland; 6 ONCOSYS Research Program in Systems Oncology, Faculty of Medicine, University of Helsinki, Helsinki, Finland

**Keywords:** MT: regular issue, oncolytic virus, adenovirus, immunotherapy, T cell exhaustion, interleukin 7, cytokine, checkpoint inhibitors, immune checkpoint blockade, renal cell carcinoma, cancer

## Abstract

Elevated levels of immune infiltration found in renal cell carcinoma are often associated with poor patient prognosis, likely due to T cell exhaustion and an immunosuppressive tumor microenvironment, which limits the efficacy of current immunotherapies. In this study, we focused on the use of Ad5/3-E2F-d24-hIL7 (TILT-517), an oncolytic adenovirus expressing interleukin-7, as a strategy to selectively lyse tumors. This approach also seeks to activate infiltrating immune cells, thereby reprogramming the immune landscape characteristic of renal cell carcinoma tumors. Furthermore, we evaluated the combination of TILT-517 with immune checkpoint inhibitors, main immunotherapeutic component for this disease. Anti-tumor efficacy was significantly observed in *ex vivo* patient-derived tumors when treated with TILT-517 in monotherapy and in treatment combination. Cellular and proteomic changes were detected in the tumor microenvironment, including enhanced cytotoxicity of effector populations such as CD4^+^, CD8^+^ T cells, natural killer, and natural killer T cells. *In vivo*, we developed a novel syngeneic Syrian hamster model for renal cancer, and TILT-517+anti-PD-L1 group presented significant enhanced tumor growth control and led to an increased number of effector and antigen-presenting cells compared to anti-PD-L1 monotherapy. Hence, TILT-517 offers a promising approach for renal cell carcinoma treatment, boosting therapeutic efficacy and reshaping the tumor microenvironment.

## Graphical abstract

**Figure:**
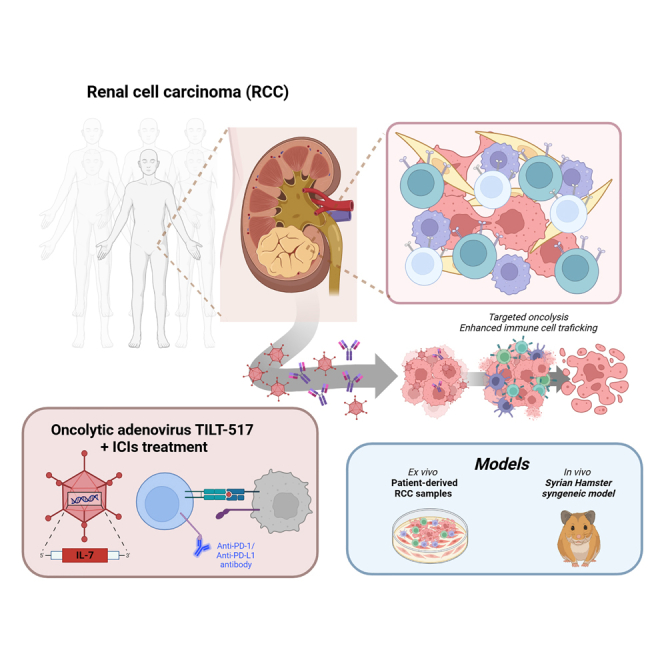

## Teaser

TILT-517 is an oncolytic adenovirus encoding interleukin-7, designed to selectively target tumor cells and stimulate the tumor microenvironment. This study confirms the potential of TILT-517, as monotherapy and in combination with checkpoint blockade for renal cell carcinoma, a disease with limited effective immunotherapy options.

## Introduction

Renal cell carcinoma (RCC) is one of the most common types of kidney cancer, amounting for around 90% of kidney malignancies.^1^ Due to the complexity and heterogeneity of this disease, the development of effective treatments remains challenging despite all the recent progresses. The immunological landscape plays a crucial role in tumor progression and treatment outcome, as increased immune cell infiltration in lesions are frequently observed in patients with poor prognosis.^2^ Targeted therapies and immunotherapies are the current standard of care to treat advanced stages of RCC (stages III and IV), which include the use of tyrosine kinase inhibitors (TKIs), such as sunitinib or axitinib, and immune checkpoint inhibitors (ICIs) such as nivolumab (anti-PD-1), and ipilimumab (anti-CTLA-4) for first-line and atezolizumab (anti-PD-L1) for second line of treatment.^3^ Further experimentation is ongoing with some recent clinical trials combining both strategies (TKI + pembrolizumab or nivolumab) or several immunotherapies (nivolumab + ipilimumab).^4^^,^^5^

ICIs function by blocking receptors that inhibit immune cells from attacking cancer cells, thus enhancing the immune response against tumors.^6^ This mechanism has demonstrated advantages in tumors with pre-existing immune infiltrate, making it a promising candidate for treating this disease.^6^ Nevertheless, ICIs’ response rate in RCC remains modest (∼20%–30%).^7^ This limited efficacy is potentially due to incomplete reversal of exhaustion, and additional contributing factors, such as low tumor immunogenicity, restricted T cell infiltration, and the prevalence of immunosuppressive elements, including regulatory T cells (Tregs) and myeloid-derived suppressor cells (MDSCs).^2^ These multifaceted challenges underscore the necessity of combinatorial strategies to achieve more robust clinical outcomes.

Oncolytic viruses (OVs) are an emerging therapeutic approach in oncology with promising efficacy in preclinical and clinical settings.^8^ OVs can selectively infect and replicate in cancer cells, leaving healthy cells unharmed.^8^ Upon infection, the release of tumor antigens and other signals by lysed cells triggers an immune response, converting an immunosuppressive into an immunostimulatory environment.^8^ This makes OVs an attractive option to overcome current limitations in RCC treatment and also to enhance existing therapies such as ICIs. Indeed, recent studies indicate a possible added benefit of combining OVs with ICIs, which could also facilitate OVs translation into clinical stages.^9^^,^^10^

The ability to insert transgenes into OVs allows targeted delivery of a desired protein directly into the tumor microenvironment (TME).^11^ Cytokines are promising transgenes for OVs, as they can boost the immune response by modulating the inflammation cascade and enhance related processes such as antigen presentation or T cell activation.^11^ Among various cytokines considered for cancer immunotherapy, interleukin-7 (IL-7) stands out due to its important role in T cell development, survival, and function.^12^ Additionally, this cytokine has demonstrated a favorable safety profile in early clinical trials, even at high doses.^13^ Precisely, IL-7 is known to be involved in several roles: enhancing T cell effector functions, preventing T cell exhaustion, promoting the expansion and survival of memory T cells and counteracting immunosuppressive mechanisms through the inhibition of Treg’s activity.^14^ Therefore, a therapy incorporating this cytokine could benefit patients with RCC.

Ad5/3-E2F-d24-hIL7 (also known as TILT-517) is an oncolytic adenovirus encoding for human IL-7 protein, which demonstrated capability to stimulate the immune response and maintain effective concentrations of IL-7 in the TME.^15^ In this study, we explore the therapeutic potential of TILT-517 for treating RCC tumors, identifying key efficacy factors, such as cancer cell killing capacity or the modulation of the TME toward a more activated state. Furthermore, we evaluate possible synergies or additive effects by combining this therapy with other immunotherapies such as ICIs, specifically with anti-PD-1 and anti-PD-L1 antibodies. To investigate this, we performed both *in vivo* approaches using a syngeneic hamster model to allow higher adenoviral permissiveness, and *ex vivo* methods with clinical samples from human RCC patients in advanced stage. Thus, highlighting its promising translation to clinical use in RCC therapy.

## Results

### Clinical RCC samples display wide heterogeneity in subtype, morphology, and phenotype

**Figure 1:**
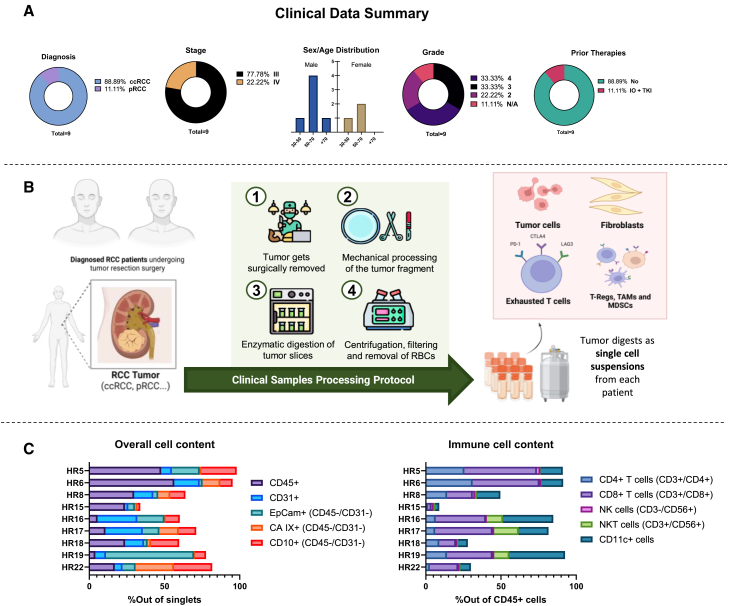
Clinical data summary and baseline status of RCC patient-derived samples (A) Chart and bar graphs detailing characteristics of the samples obtained displaying diagnosis, stage, sex/age distribution, grade, and prior therapies. ccRCC (clear-cell renal cell carcinoma), pRCC (papillary renal cell carcinoma), IO + TKI (immuno-oncology + tyrosine kinase inhibitor treatments). (B) Graphical representation of the protocol used for processing of the RCC clinical samples. (C) Percentage of CD45^+^, CD31^+^ cells, EpCam^+^, CA IX^+^, CD10^+^ tumor cells, and CD4^+^, CD8^+^, CD11c^+^, NKT (CD3^+^CD56^+^), and NK (CD3^˗^CD56^+)^ immune cells in single-cell suspensions for each of the samples.

A total of 9 RCC samples were collected and included in the study, encompassing similar variability as seen in patients in age, sex, tumor subtype, grade, and stage of the disease (Table S1). Most of the lesions (8 out of 9 cases) were diagnosed as clear cell renal cell carcinoma (ccRCC), while one specimen was diagnosed as papillary renal cell carcinoma (pRCC) (Figure 1A). Most of the samples had a stage III designation (78% of total), followed by a few cases in stage IV (22% of total) (Figure 1A). The majority of the patients were between 50 and 70 years old (6 out of 9 cases), and males cases were more common than females (6 out of the total cases) (Figure 1A). Tumor grade was ranging between a few cases of grade 2 (22% of total), followed by majorly 3 and 4 (33% each) (Figure 1A).

Regarding prior cancer therapies, only one patient received previous treatment with immune-oncology treatment (checkpoint inhibitors) and sunitinib (tyrosine kinase inhibitor, targeted therapy) (Figure 1A). For treatment efficacy and immunological studies, samples were processed to single-cell suspensions using an established protocol (Figure 1B). Antibody staining confirmed that the samples displayed a wide range of immune infiltrate levels (CD45^+^ cells), endothelial cells (CD31^+^) and cancer cell markers (CA IX, CD10, and EpCam), indicating variability in tumor cell profiles within the collected samples (Figure 1C). Also, the main immune populations such as CD8^+^ and CD4^+^ T cells, NK cells (CD3-CD56^+^), NKT cells (CD3^+^CD56^+^), and CD11c^+^ cells were identified, with general variability in their proportions (Figure 1C).

### TILT-517 monotherapy and in combination with anti-PD-1/anti-PD-L1 promote tumor cell killing in RCC samples compared to ICI monotherapy treatment conditions

**Figure 2:**
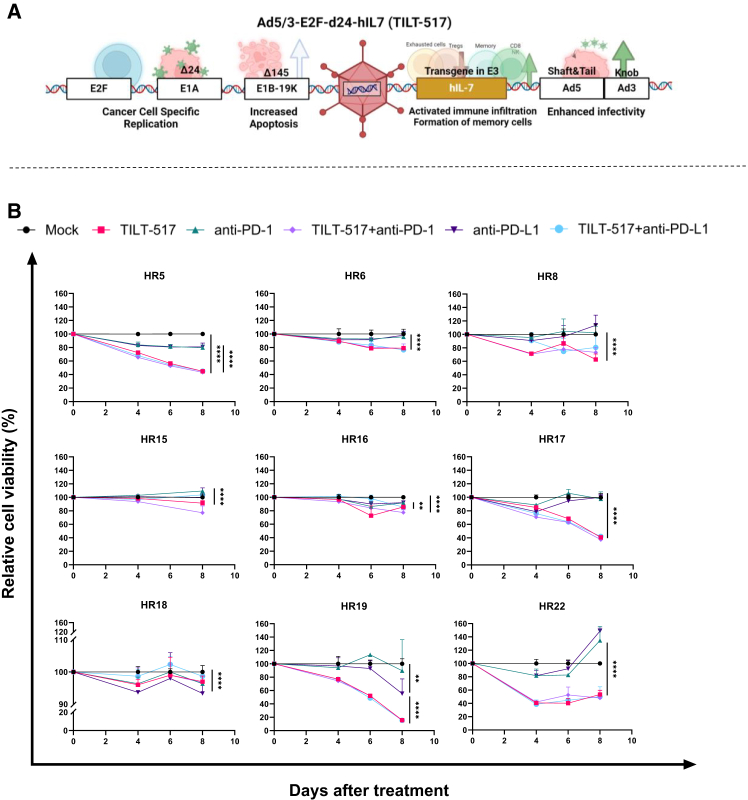
Cancer cell killing study of RCC tumor digestions treated with Ad5/3-E2F-d24-hIL7 (TILT-517) and/or in combination with anti-PD-1 or anti-PD-L1 (A) Scheme of the structure of Ad5/3-E2F-d24-hIL7 adenovirus (TILT-517). (B) Relative human cancer cell viability after exposing the samples to 100 VP/cell of TILT-517 or 0.2 mg/mL of anti-PD-1 or anti-PD-L1. Experiments were performed in triplicates, and data were normalized to the untreated condition (mock). All data are presented as mean (SD). For the cell viability comparisons, two-way ANOVA Tukey multiple comparisons test was used. ∗∗∗∗*p* < 0.0001, ∗∗*p* < 0.01.

To evaluate the lytic capacity of TILT-517 alone (structure can be found in Figure 2A) or in combination with ICIs, tumor single-cell suspensions from these 9 patients were treated *ex vivo* with either TILT-517 and/or either anti-PD-1 or anti-PD-L1 (Figure 2B). Overall, all samples displayed a reduction in cell viability in the TILT-517 treated groups compared to mock and to ICI monotherapy conditions. We observed notably significant results between mock and viral conditions (*p* < 0.0001) in 7 out of 9 samples (78% of total) and varied but still significant differences between ICI monotherapies and viral conditions (*p* < 0.01 in HR16; *p* < 0.0001 in all the other samples) in 8 out of 9 samples (89% of total). There was only one case (HR18) where ICI monotherapy was similar in efficacy compared to the virus, yet still substantial changes compared to mock were observed (*p* < 0.0001). Non-significant differences were observed between TILT-517 monotherapy and its respective combinations with anti-PD-1 or anti-PDL-1, thus indicating that the combination treatments neither enhanced nor reduced the tumor cell killing effect. qPCR confirmed a statistically significant increase in expression of adenovirus hexon gene in the infected groups (*p* < 0.001) (Figure S1). No correlations were found between these results and the different cell population abundance obtained at the baseline cell study, nor the patient clinical data.

### Effector CD8^+^ and CD4^+^ lymphocyte populations are enhanced in RCC samples treated with TILT-517 and its combination groups

**Figure 3:**
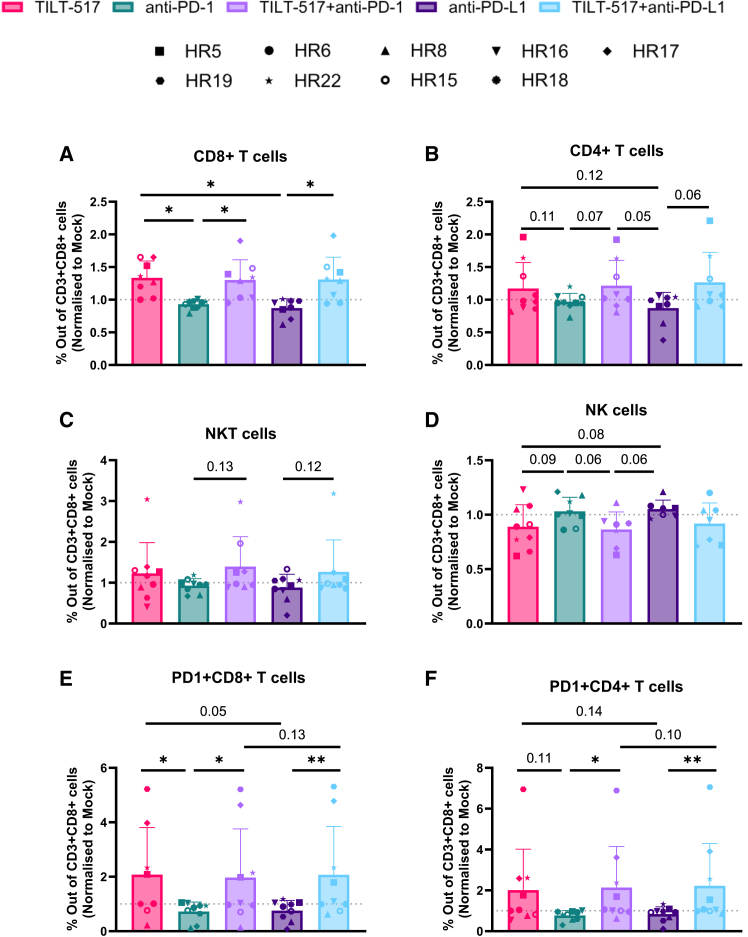
Observed TME changes in RCC samples after 5 days of treatment. Comparison of the differences among the main populations between treatment conditions Results from flow cytometry targeting (A) CD8^+^ T cells, (B) CD4^+^ T cells, (C) NKT cells (CD3^+^CD56^+^), (D) NK cells (CD3^˗^CD56^+^), (E) PD1^+^ CD8^+^ T cells, and (F) PD1^+^ CD4^+^ T cells. Samples were treated with 100 VP/cell of TILT-517 and/or 0.2 mg/mL of anti-PD-1 or anti-PD-L1, incubated for 5 days, and stained (300 K events were detected). Data were normalized to the untreated condition (mock); each point corresponds to a sample value obtained pooled from a previous triplicate experiment. All data are presented as mean (SD). Paired t tests and Wilcoxon paired test were used for comparing all the different treatment conditions, and *p* values under 0.15 were displayed. ∗∗*p* < 0.01, ∗*p* < 0.05.

Cellular changes in the TME were studied in all samples after 5 days of incubation with either monotherapies or combination treatments (Figures 3A–3F). Individual sample results were normalized to their respective mock values and subsequently pooled to assess overall variations. The largest differences were noticed in the CD8^+^ T cell subset, showing increased presence in groups treated with TILT-517 compared to the non-TILT-517 treated groups (Figure 3A). A statistically significantly higher presence was observed in TILT-517-treated group compared to anti-PD-1 or anti-PD-L1 groups (*p* = 0.0049, *p* = 0.0149, respectively), and combinations against their respective ICI monotherapies (*p* = 0.0185 anti-PD-1, *p* = 0.0493 anti-PD-L1). Despite not reaching statistical significance, a similar trend was observed in CD4^+^ T cells and NKT cells, where TILT-517 monotherapy resulted in a higher percentage than ICI monotherapies in those subsets, and combination groups displayed higher percentages compared to their respective ICI monotherapy (*p* values ranging from *p* = 0.06 to *p* = 0.13) (Figures 3B and 3C). Analysis of NK cells revealed a close-to significant trend displaying lower percentages in TILT-517-treated compared to ICI groups: TILT-517 vs. anti-PD-1/anti-PD-L1 (*p* = 0.09, *p* = 0.08), TILT-517 + anti-PD-1 vs. anti-PD-1 (*p* = 0.06) and TILT-517 + anti-PD-L1 vs. anti-PDL-1 (*p* = 0.16) (Figure 3D). Finally, the study of exhausted T cells demonstrated that tumors treated with TILT-517 displayed higher percentages compared to the ICIs monotherapies (Figures 3E and 3F). PD1^+^CD8^+^ T cells presented higher significant differences when comparing TILT-517 vs. anti-PD-1 (*p* = 0.015) and anti-PD-L1 (*p* = 0.05), and similarly for TILT-517 + anti-PD-1 vs. anti-PD-1 (*p* = 0.0391), and TILT-517 + anti-PD-L1 vs. anti-PD-L1 (*p* = 0.0039) (Figure 3E). A similar observation was made when analyzing changes in PD1^+^ CD4^+^ T cells (TILT-517 + anti-PD-1 vs. anti-PD-1 *p* = 0.0156, TILT-517 + anti-PD-L1 vs. anti-PD-L1 *p* = 0.0039) (Figure 3F). Thus, TILT-517, either in monotherapy or in combination, can modulate the lymphocyte levels and specially increase the number of CD8^+^ T cells found in the TME.

### Increased abundance of effector cytokines is connected to improved tumor cell killing capacity

**Figure 4:**
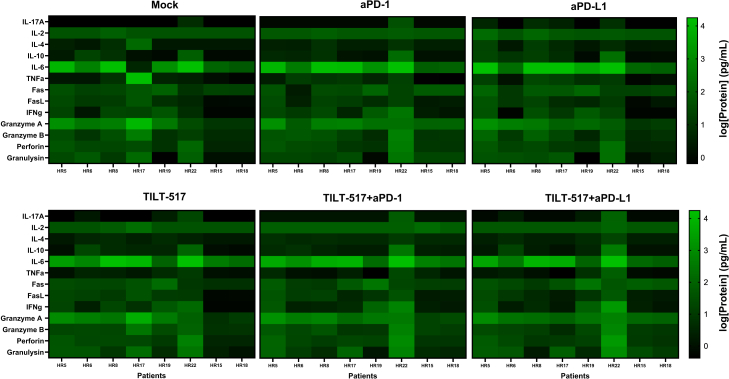
Cytokine expression differences across RCC patient samples after 5 days of treatment Supernatant from the treated samples was analyzed for several cytokines related to the inflammation and activation status for mock, anti-PD-1, anti-PD-L1, TILT-517, TILT-517 + anti-PD-1, and TILT-517 + anti-PD-L1. Experiment was run in duplicates, and data were obtained as absolute protein concentration from LEGENDPLEX Suite (BioLegend). Log transformed values were plotted as a heatmap plot.

To evaluate possible inflammatory changes and their implication in the TME of the treated samples, a set of immunomodulatory cytokines was evaluated in supernatants collected from the tumor single-cell suspensions on day 5 of the *ex vivo* treatment (Figure 4). Results revealed a clear increase in expression of IL-6, TNFa, Fas and FasL, IFNg, granzyme A, granzyme B, perforin, and granulysin in 7 out of 8 samples (78% of total). The only exceptions were HR15 and HR18, samples with the lowest cell-killing profile in the cell viability assay. On the contrary, HR22 displayed high values within all the cytokines analyzed compared to the rest of the samples. No significant differences were observed when comparing between treatment conditions (Figure S2A). hIL-7 protein levels were also analyzed, revealing significantly higher values in virus-treated conditions, likely due to transgene expression (Figure S2B).

### TILT-517 modulates regulatory, naive, and memory T cells

**Figure 5:**
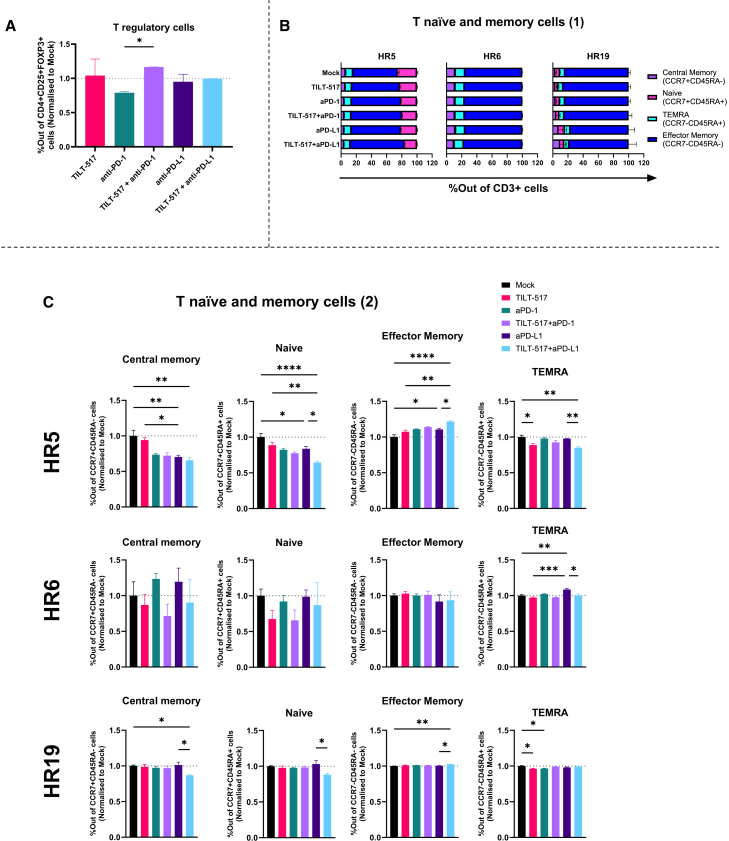
Modulation of special population subsets based on treatment condition (A) Presence of regulatory T cells (FOXP3^+^CD25^+^CD4^+^), with data normalized to untreated (mock) condition. (B) Schematic representation of the distribution for T memory cell populations in a flow cytometry plot based on C-C chemokine receptor type 7 (CCR7) and CD45RA expressions. (C) T naive and memory cells in HR5, HR6, and HR9 samples. Stacked barplots display the distribution for each subset (central memory, naive, TEMRA, and effector T cells) on the mentioned samples, and bar-plots below display comparisons based on treatment for the same subsets studied. Experiments were run in triplicates, and all data are presented as mean (SD). Statistical significance was tested using paired t tests and one-way ANOVA with Tukey multiple comparisons tests. ∗∗∗∗*p* < 0.0001, ∗∗*p* < 0.01, ∗*p* < 0.05.

Flow cytometry was performed to assess treatment effects on Tregs and naive and memory T cells. For T regs, the combination of TILT-517 + anti-PD-1 displayed statistically significant higher values compared to anti-PD-1 monotherapy (*p* = 0.023) (Figure 5A). Memory and naive T cells were assessed on HR5, HR6, and HR19 through four different populations: central memory (CM, CCR7^+^CD45RA^–^), naive (N, CCR7^+^CD45RA^+^), effector memory (EM, CCR7^-^CD45RA^-^), and effector memory re-expressing CD45RA (TEMRA, CCR7^-^CD45RA^+^). Results demonstrated heterogeneity between samples, being EM T cells were the largest memory cell subset ranging from 60% to 90%, while naive, CM, and TEMRA cell values remained in lower presence (Figure 5B).

Differences by treatment condition were also varying on the sample, being clearly observable in HR5, with lower levels of central memory, naive T, and TEMRA cells in TILT-517-treated conditions compared to ICIs monotherapy and untreated group (Figure 5C). TILT-517 + anti-PD-L1 significantly reduced naive T cells compared to all other conditions (*p* = 0.0405 to *p* < 0.0001), and similarly for CM and TEMRA cells compared to anti-PDL-1 and untreated (*p* < 0.01 in average). On the contrary, effector memory cells displayed higher presence in both combination groups, being significantly higher compared to the untreated group (*p* = 0.0022 in TILT-517 + anti-PD-1, *p* < 0.0001 in TILT-517 + anti-PD-L1).

### Increased cancer cell killing correlates with high lymphocyte and effector cytokine levels in RCC tumors treated with TILT-517

To understand the contribution of tumor-infiltrated immune cells and cytokine production generated by the therapy to cancer cell cytotoxicity, correlations were conducted using data from immune cell profiling, cytokine quantification, and cell viability studies from the TILT-517-treated groups.

**Figure 6:**
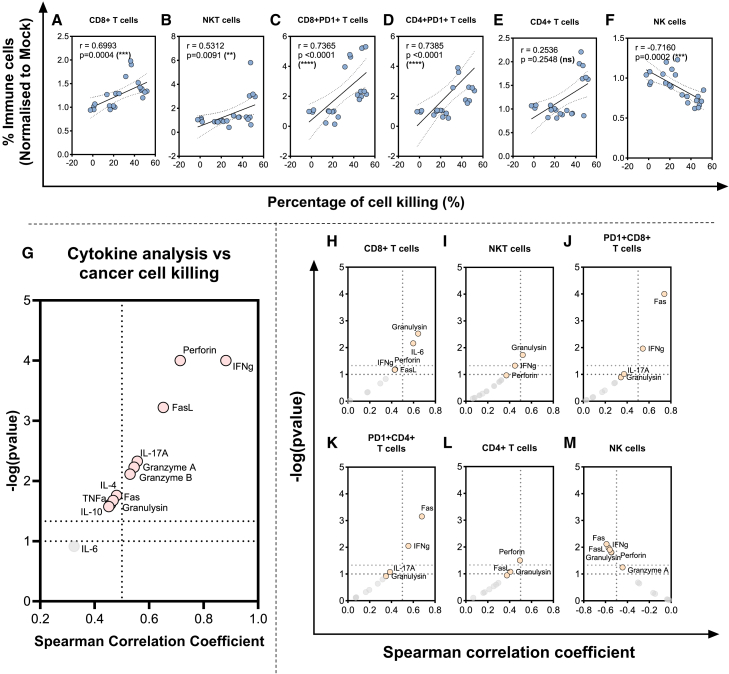
Correlations between percentage of cell killing, immune cell infiltration, and cytokine expression levels Associations of the killing capacity results at day 6 from viral treated groups with the presence at day 5 of (A) CD8^+^ T cells, (B) NKT cells (CD3^+^CD56^+^), (C) CD4^+^ T cells, (D) NK cells (CD3^˗^CD56^+^), (E) CD8^+^PD1^+^ T cells, and (F) PD1^+^CD4^+^ T cells. (G) Volcano plot with multiple correlations between cell killing percentage at day 6 and cytokine analysis values at day 5. Multiple volcano plots were also plotted to express the correlation results between cytokines and immune cell presence: (H) CD8^+^ T cells, (I) CD4^+^ T cells, (J) NKT cells (CD3^+^CD56^+^), (K) NK cells (CD3^˗^CD56^+^), (L) CD8^+^PD1^+^ T cells, and (M) PD1^+^CD4^+^ T cells. Correlations were performed using Spearman correlation method. ∗∗∗∗*p* < 0.0001, ∗∗∗*p* < 0.001, ∗∗*p* < 0.01, ns = non-significant.

CD8^+^ T cells, NKT cells, CD8^+^PD1^+^, and CD4^+^PD1^+^ T cells displayed a significant positive correlation with cell killing (*p* < 0.001, *p* = 0.0091, *p* < 0.0001, *p* < 0.0001 respectively), and CD4^+^ T cells did not have significant correlation (Figures 6A–6E). Interestingly, NK cell levels negatively correlated with improved cell killing (*p* < 0.0001) (Figure 6F).

In terms of cytokine production, strong correlations in most of the analytes were observed (Figure 6G). Overall, cytokines associated with effector lymphocyte cytotoxicity response (IFNg, perforin, granzyme A, granzyme B, and granulysin), cell death (FasL and TNFa), and IL-17A showed strong positive correlation with cancer cell killing (*p* = 0.025 to *p* < 0.0001). Finally, the different immune cell subtypes were correlated with the cytokine production. Granulysin was found positively correlated with CD8^+^ T cells and NKT cells (*p* = 0.03, *p* = 0.018, respectively) (Figures 6H and 6I). Fas and IFNg correlated with PD1^+^CD8^+^ (*p* < 0.0001 and *p* = 0.0099) and PD1^+^CD4^+^ T cells (*p* < 0.001, *p* = 0.0089), and perforin with CD4^+^ T cells (*p* = 0.03) (Figures 6J–6L). Curiously NK cells had a strong negative correlation for several pro-inflammatory cytokines: Fas (*p* = 0.007), FasL, IFNg, perforin, and granulysin (*p* = 0.01 to 0.015) (Figure 6M). No correlations were found between lymphocyte nor cytokine levels and the different cell population abundance at baseline, nor the patient clinical data.

### TILT-517 promotes tumor growth control and infiltration of effector cells in a newly stablished RCC Syrian hamster model

**Figure 7:**
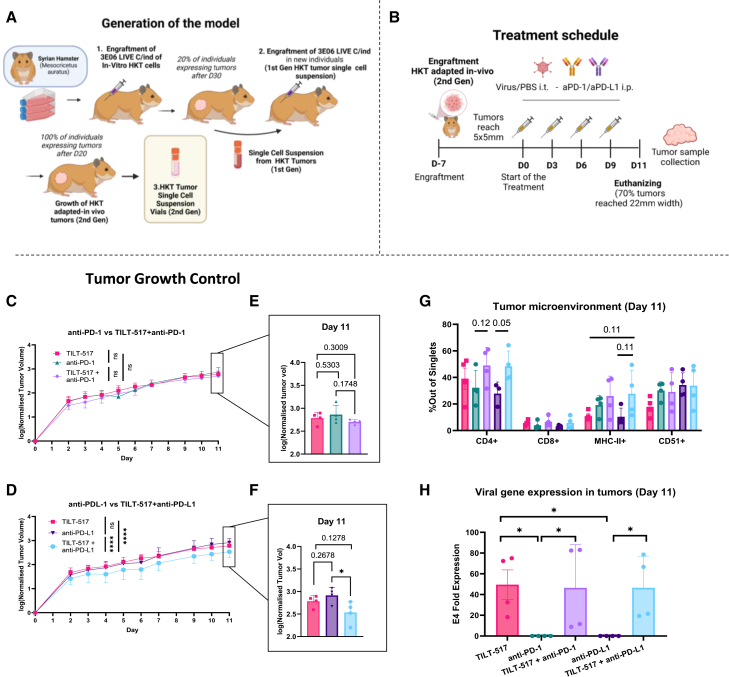
Generation of a renal cancer hamster model and treatment with Ad5/3-E2F-d24-hIL7 (TILT-517) + anti-PD-1/anti-PD-L1 (A) Schematic representation of the generation of a stable renal cancer model in Syrian hamster for the study of oncolytic adenovirus. (B) Schematic diagram for the testing of TILT-517 in combination with anti-PD-1 or anti-PD-L1, after achieving the adapted-in vivo HKT-1097 cell line for engraftment. Tumor growth control curves comparing monotherapy and combination of TILT-517 with (C) anti-PD-1 and (D) anti-PD-L1, based on the log values of tumor volume on each individual and normalized at the start of the treatment. In-depth comparison between these groups on day 11 of treatment were performed for (E) antiPD-1 and (F) anti-PD-L1. (G) Study of the tumor microenvironment on day 11 of treatment using tumor samples from each group individual measuring for the presence of CD4^+^, CD8^+^, MHC-II^+^, and CD51^+^ cells. (H) Verification of TILT-517 viral replication within the tumors on day 11 based on E4 expression. Each point corresponds to one individual, and all data are presented as mean (SD). Two-way ANOVA with Bonferroni multiple comparisons test was used to assess overall tumor growth, and paired t test was used for evaluating differences on day 11. Statistical significance in viral replication and tumor microenvironment studies was determined by unpaired t test and Mann-Whitney test.∗∗∗∗*p* < 0.0001,∗*p* < 0.05, ns = non-significant.

To generate a syngeneic hamster model for RCC, HKT-1097 was chosen due to its permissivity for human adenovirus (in this case, TILT-517) infectivity, cell lysis, and transgene production (Figure S3A). An initial *in vivo* adaptation experiment was conducted to optimize HKT-1097 cell growth in the *in vivo* model and improve the engraftment rate, as previously described (Figure 7A), and HKT-1097 still maintained these previously assessed properties after the process (Figure S3B). Subsequentially, an experiment was conducted to assess the potential benefits of combining TILT-517 with ICIs in a clinically relevant model permissive to TILT-517 infection and oncolysis (Figure 7B). Tumor growth curves showed improved tumor control for TILT-517 + anti-PD-1 or anti-PD-L1 compared to their ICIs monotherapy alternatives (Figures 7C and 7D), and the combination with anti-PD-L1 displayed highly significant differences (*p* < 0.0001). Pairwise comparisons from tumor volumes on day 11 (last day of experiment) also proved differences in tumor size between groups in both anti-PD-1 vs. TILT-517 + anti-PD-1 (*p* = 0.17) (Figure 7E) and anti-PD-L1 vs. TILT-517 + anti-PD-L1 (*p* = 0.00456) (Figure 7F). Groups treated with the TILT-517 virus showed an increased trend in the number of CD4^+^ T, CD8^+^ T, and MHC-II^+^ cells infiltrating tumors, and a decrease in CD51^+^ endothelial cells, with major differences for anti-PD-L1 comparison (Figure 7G). qPCR for adenoviral E4 gene confirmed the infection of the tumors of the viral treated groups, compared to respective non-TILT-517-treated groups (both *p* = 0.0286) (Figure 7H).

## Discussion

The main scope of this study was to evaluate the efficacy of an oncolytic adenovirus encoding for human interleukin-7, Ad5/3-E2F-d24-hIL7 (TILT-517), as a treatment for RCC, and its combination with ICIs. To achieve that, a human *ex vivo* model and a novel hamster *in vivo* model were used in the study.

We acquired nine fresh kidney cancer samples from patients with varying clinical diagnoses, each one presenting distinct immunological and cellular heterogeneity. This highlights some of the challenges immunotherapies must overcome for successful patient response in the clinic. Concurrently, this also allowed us to evaluate viral efficacy considering factors as tumor and immune cell density, potential immunosuppressive components, or the proportions of epithelial and endothelial tissues within each sample. In the context of TILT-517 therapy, baseline tumor variation did not represent an impediment to anti-tumor response, and the virus performed equally well in advanced-stage patients—the desired target for our therapy.^3^

TILT-517-treated groups showed superior tumor cell killing compared to the ICI monotherapies and untreated group, with marked differences in 78% of the samples. In the clinical context, these results become more relevant as the used ICIs, anti-PD-1 and anti-PD-L1, which represent the closest immunotherapeutic approach used in standard care, exhibit an average efficacy of only 30%.^7^ In our results, we did not observe the added benefit of combining ICIs with the TILT-517 treatment, as no apparent additive or synergistic effect was noted in the tested conditions. Altogether, this may suggest that the viral therapy itself is the primary driver of the response, rather than a cumulative cytopathic effect.

Besides direct cancer cell killing, the engagement of effector lymphocytes CD8^+^ T, CD4^+^ T, and NKT cells and the presence of associated proinflammatory molecules in the TME suggests that TILT-517 efficiently triggered elements of innate and adaptive immune response. This proposes that TILT-517 can disrupt the silenced and suppressed state of the TME commonly observed in RCC, effects that are not often achieved with ICI monotherapies.^2^ In fact, the correlations observed support this hypothesis, as a higher presence of these lymphocytes and proinflammatory molecules seems to be connected to improved cancer cell killing.^2^^,^^16^ Overall, this would indicate a shift of the TME toward a more functional immune state, suggesting improved outcomes for patients with otherwise unfavorable prognostic features.

Alternatively, NK cells seem to deviate from these trends, which could be potentially explained by two fronts. Firstly, NK cells provide an antiviral defense against TILT-517 due to their function in targeting viral infections. This would reduce the effective viral load, thereby restricting the widespread tumor cell killing by oncolysis.^17^ However, an alternative explanation could be that the observed results reflect a late-stage effector situation. As earlier indicated, NK cells represent one of the first barriers to immune effector cells. Thus, the findings on day 5 may indicate a scenario when the peak for the NK cell response is long gone.^18^ Prior studies with OVs have indicated that NK cells can benefit OV treatments rather than oppose them, which means that NK cell responses and OV therapy are not inherently antagonistic but may depend on factors, such as timing, dosage, and treatment context.^19^ This argument is also supported by the presence of upregulation of exhausted PD1^+^ lymphocytes and their correlation with cancer cell killing, suggesting that analysis on day 5 could be reflecting the end of the response when the immune cells are returning to a non-activated state.^20^

As noted earlier, TILT-517 drives inflammation within the TME, underscoring a diverse range of cell death pathways: IFNg and granzymes A-B promote tumor cell apoptosis through cytotoxic T cells, Fas-FasL and perforin indicate active immune-mediated tumor cell killing via apoptosis, and granulysin levels reinforce this cytotoxic activity through membrane disruption.^21^^,^^22^ Correlations with IFNg, Fas, perforin, and granzymes A-B were highly statistically significant than the other molecules, potentially indicating an important role in the mechanism of action of the therapy. The absence of major differences across treatment conditions in the cytokine analysis reinforces this later-stage activation hypothesis, coupled with the short half-life of these proteins.^23^

Initially, we hypothesized that the use of ICIs could mitigate the exhaustion process to ideally promote a long-lasting response.^6^ *Ex vivo*, we observed differences between anti-PD-1 and anti-PD-L1-treated groups when compared to their monotherapies, with anti-PD-1 potentially demonstrating a modest effect in alleviating T cell exhaustion. This may be attributed to its mechanism of action, involving direct binding and interaction with PD-1 on immune cells, whereas anti-PD-L1 might exert effects indirectly.^24^ Regarding the effects on the IL-7 transgene, we observed that Treg levels were elevated in TILT-517-treated groups, which initially would relate to the reduction of the inflammatory state after the effective cell killing. Nevertheless, beneficial implications of these observations should also be considered, as recent studies have identified advantageous features associated with specific populations of Tregs.^25^ The study of memory and naive compartments revealed a predominance of T effector cells overall and a decrease of T naive and central memory cells after treatment. Again, this shift may reflect immune cell activation and differentiation processes occurring within the *ex vivo* culture system, potentially mimicking priming events.^26^^,^^27^

Although *ex vivo* models provide quite approachable characterization of the TME, they do lack the capacity to replicate a functioning immune system that responds dynamically to the therapy. In the present work, the *ex vivo* results were nicely complemented by an *in vivo* model. Tumor growth curves demonstrated an improvement specially when TILT-517 was combined with anti-PD-L1, compared to anti-PD-L1 and virus monotherapy, yielding statistically significant results. This may be attributed to the fact that the anti-PD-L1 used was specific for Syrian hamster, whereas the anti-PD-1 was initially designed for mouse but exhibited hamster cross-reactivity.^28^ This lack of specificity may have affected the therapeutic potency of this model. Despite observing an increase in TILT-517-treated groups, no significant differences were observed in the analyses of intratumoral immune cells. Nevertheless, this modest trend could be mirroring the situation observed in the *ex vivo* setting and associated with the *in vivo* mechanism of action of the therapy.

Further investigation toward clinical stages should be performed, as several limitations must be acknowledged. More comprehensive understanding of the mechanisms of action of TILT-517 in this context is essential to better define the immune pathways complementing the oncolysis process, as these findings may be influenced by the limitations inherent in both *ex vivo* and *in vivo* models used.^29^ Using more direct methods to separate tumor and immune cell populations *ex vivo* could provide more specific viability results, minimizing potential interferences from current viability assays (e.g., MTS, which primarily reflects metabolic activity) and allowing for a clearer link to immune characterization. Another potentially effective approach could involve utilizing alternative *in vivo* animal models, such as mice or rats, to emphasize response studies rather than focusing solely on oncolysis, and with higher reagent diversity available.^30^ It could also be valuable to investigate the spatial distribution of each cell type and identify detailed changes in the overall immune landscape, as these factors may provide further insights into the therapy’s effects on the tumor microenvironment. Ultimately, a study to identify proportion of T cells responding to viral versus tumor antigens could be helpful to observe if the treatment can generate long anti-tumor response, or if it is merely relying on the response based on antiviral mechanism. Nevertheless, studying this can be tricky, as sequencing can lead to weak TCR identification due to the presence of neoantigens presented after tumor lysis, and since you would need to determine the specificity anyway.

Overall, our data demonstrate that the proposed adenovirus encoding IL-7 effectively reduces tumor burden and stimulates the presence of effector immune cells, including CD4^+^, CD8^+^, NKT, and NK cells, marking it as a promising therapeutic option for advanced and metastatic RCC and suitable for combination with ICIs such as anti-PD-1 or anti-PD-L1. These findings highlight the potential of this therapeutic strategy to significantly improve outcomes for patients with this disease and support further research into the use of oncolytic viruses in cancer immunotherapy.

## Materials and methods

### Ethics statement

All animal experiments described in the paper were approved by the Provincial Government of Southern Finland and the Experimental Animal Committee of the University of Helsinki (license no. ESAVI-26562-2022). Cancer samples were collected from the patients undergoing surgical resection at the Helsinki University Central Hospital (HUS, Helsinki, Finland). Sample collection was approved by HUS Operatives Ethics Committee (permit no. HUS/325/2023) and the patients gave their written consent.

### Virus construction

The Ad5/3-E2F-d24-hIL7 (TILT-517) virus was constructed previously.^31^ Tumor-specific replication was achieved by two modifications: an E2F promoter and a 24- base pair deletion in the constant region of E1A, which determines tumor selectivity regarding viral replication. Human IL7 (hIL7) coding sequence (GenBank: NP_000871.1) was introduced in E3 region replacing gp19k and 6.7k genes via bacterial artificial chromosome (BAC) recombineering strategy.^32^ The resulting viral vector sequence was confirmed by next-generation sequencing.

### Patient-derived samples collection and processing

Tumor samples were collected from patients with RCC confirmed diagnosis (Table S1) who underwent surgical resection at the Hospital District of Helsinki and Uusimaa (HUS). A total of 9 samples were received and processed upon arrival to obtain single-cell suspensions, using a previously described protocol, and selected by viability.^33^ In short, tumor samples were sliced into small fragments of around 5 mm size and incubated in RPMI 1640 media supplemented with 1% L-glutamine, 1% Pen/Strep, 10% fetal bovine serum (FBS), collagenase type I (170 mg/L), collagenase type IV (170 mg/L), DNase I (25 mg/mL), and elastase (25 mg/mL) (Worthington Biochemical, NJ, USA) for a 3-h enzymatic digestion with rocking at +37°C. After digestion, the suspension was treated with ACK lysis buffer (Sigma-Aldrich, MO, USA) and filtered through a 70 μm filter to remove undigested fragments, debris, and red blood cells. The final single-cell suspensions were frozen in 10% DMSO freezing media and stored at −140°C until further use.

### Cell viability assays

Single-cell suspensions were thawed and plated in a seeding density of 3 × 10^5^ viable cells/well in triplicates in a 96-well plate (U-Bottom), in DMEM growth supplemented with 10% FBS, 1% Pen/Strep, 1% L-glutamine. Cells were treated with either TILT-517 (100 VP/viable cell), anti-PD-1 (pembrolizumab, Merck, NJ, USA, 0.2 mg/mL), anti-PD-L1 (atezolizumab, Roche, Basel, Switzerland, 0.2 mg/mL) alone, or the combination of thereof. Cell viability was assessed on days 4, 6, and 8 using 20% of CellTiter 96 AQueous One Solution Proliferation Assay reagent (Promega, WI, USA) according to the manufacturer’s instructions. Absorbance was recorded at 490 nm using Hidex Sense reader (Hidex, Turku, Finland). Data were normalized to the untreated mock group from the same day.

### Flow cytometry

To characterize the immune status of the single-cell suspensions at baseline in the collected RCC tumor samples, a flow cytometry run was performed to evaluate the expression of the following markers: CD45, CD8, CD4, CD56 for immune cells; CD31 for endothelial cells and CA IX, CD10, and EpCam for tumor cells.

For the flow runs assessing immunological changes on day 5 based on treatment condition, PD-1 marker was also used. For the flow cytometry panels evaluating naive and memory compartments, antibodies against CD45RA and CCR7 markers were used. Samples were Fc blocked prior to staining with Human TruStain FcX Receptor Blocking Solution (Biolegend, CA, USA).

For the immune studies related to the syngeneic hamster model, the single-cell suspensions were treated with a 1:1 ratio mix of Human TruStain FcX Receptor Blocking Solution (Biolegend, CA, USA) and Purified Rat Anti-Mouse CD16/CD32 blocking solution (BD Biosciences, NJ, USA), and stained with antibodies targeting CD8^+^, CD4^+^, MHC-II^+^, and CD51^+^ cells.

Regulatory T cells presence was analyzed using eBioscience Human Regulatory T cell Staining Kit (ThermoFisher, MA, USA), according to manufacturer’s instructions.

A list of all antibodies used in this study as well as a distribution of every panel, are presented in Table S2. Fluorochrome compensation was performed before flow runs with UltraComp eBeads Compensation Beads (ThermoFisher, MA, USA). Fluorescence was acquired using NovoCyte Quanteon Flow Cytometer Systems (Agilent, CA, USA) with at least 300 k or 100 k events collected per well. Data were subsequently analyzed with FlowJo v.10.9.1 (FlowJo LLC, OR, USA).

### Quantitative PCR

Infected RCC single-cell suspensions from the *ex vivo* samples and tumor cells harvested on Day 10 from the *in vivo* study were collected for detection of viral gene expression. DNA was extracted from the samples using the QIAamp DNA Mini Kit (51306, QIAGEN, Hilden, Germany) following the manufacturer’s instructions. The purified DNA was quantified to check the relative expression of adenovirus *hexon* and *E4* genes, using Light Cycler Probes master mix (Roche, Basel, Switzerland). The total gene expression was normalized to human β-actin or hamster γ-actin housekeeping gene, respectively. All PCR reactions were run in triplicates. A list of all primers used in the study can be found in Table S3.

### Chemokines and cytokines analysis

Supernatants from *ex vivo* samples were collected on day 5 after treatment and the expression of a range of cytokines and chemokines were measured using the Human CD8 and NK Response LEGENDplex panel (BioLegend, CA, USA), according to the manufacturer’s instructions. Samples were measured in duplicates using with NovoCyte Quanteon Flow Cytometer Systems (Agilent, CA, USA) and analyzed with LEGENDplex Data Analysis Software Suite (BioLegend, CA, USA).

Human IL-7 concentration was measured from the same supernatants using an IL-7 Human ELISA Kit (Thermo Fisher Scientific, MA, USA), according to the manufacturer’s instructions, and absorbance was recorded using Hidex Sense reader.

### Virus permissiveness and human IL-7 expression analysis

The Syrian hamster cancer cell line HKT-1097 (renal carcinoma) was obtained from Leibniz Institute (DSMZ, Braunschweig, Germany) and cultured under recommended conditions. HKT-1097 (prior and after *in vivo* adaptation) was infected with a range of different virus concentrations (0, 10, 100, 1 k, 5 k, or 10 k VP/cell) of TILT-517. On days 3, 5, and 8, cell viability was measured using 20% of CellTiter 96 AQueous One Solution Proliferation Assay reagent (Promega, WI, USA), recording the absorbance at 490 nm with Hidex Sense reader (Hidex, Turku, Finland). Data were normalized to the untreated mock control group.

Human IL-7 concentration was measured from day 3 cell culture supernatants from samples infected with 1,000 and 10,000 VP/cell of TILT-517 using an IL-7 Human ELISA Kit (Thermo Fisher Scientific, MA, USA), according to the manufacturer’s instructions, and absorbance was recorded using Hidex Sense reader.

### HKT-1097 cell line expansion *in vivo*

To study the *in vivo* therapeutical potential of TILT-T517 in a relevant RCC pre-clinical model, Syrian golden hamsters (*Mesocricetus auratus*, Envigo, IN, USA) were selected for being a semi-permissive species to human adenovirus replication that respond to human cytokines, including IL-7.^15^ To establish a robust cell line capable of forming tumors in hamsters and enhancing the engraftment rate, multiple *in vivo* passages were conducted to facilitate adaptation and ensure consistent tumorigenicity in the host species.

A total of 10 male 5-week-old animals were subcutaneously implanted with 3 × 10^6^ HKT-1097 cells into their right flank. Tumors were allowed to grow for 30 days, when animals were euthanized as the tumor reached 22 mm in width. Tumors were collected, processed, and subsequently engrafted into new 10 naive animals. Briefly, tumors were digested and filtered through a 70 μm filter following ACK buffer incubation to remove red blood cells. A second round of engraftment was performed using 3 × 10^6^ cells from the first round, per animal. Tumor growth was monitored and tumors were collected after euthanizing on day 20 under the same criteria and processed using the same protocol as for the first *in vivo* passage. Cells were frozen using freezing media with 10% DMSO and stored up to −140°C upon further use.

### Syngeneic RCC hamster experimental model

To assess the efficacy of the combination treatment in the new developed model, 16 male 5-week year old animals were engrafted subcutaneously with 3 × 10^6^ HKT-1097 *in-live* cells into the right flank and randomized into five treatment groups (TILT-517, anti-PD-1, anti-PD-L1, TILT-517+anti-PD-1, TILT-517+anti-PD-L1), 4 individuals each after tumors reached 5 to 6 mm in diameter. TILT-517 was administered intratumorally with 1 × 10^9^ VP per injection, and anti-PD-1 (anti mouse PD-1 clone RMPI-14, BioXcell, NH, USA) or anti-PD-L1 (Syrian hamster anti-PD-L1 clone 11B12-1, TILT Biotherapeutics Ltd, Helsinki, Finland) was administered intraperitoneally with 0.1 mg/injection. Tumors were measured every other day with a digital caliper, and tumor volumes were calculated as (length x width^2^)/2. All hamsters received at least a total of four rounds of treatment before they were euthanized on day 11, when tumors achieved a size higher than 22 cm in width. Tumors were collected and processed into single-cell suspensions through 70-μm filtering and stored at −80C for further analysis. Tumor individual growth curves can be found in Figures S4A and S4B.

### Statistical analysis

GraphPad Prism v.10.1.2 (GraphPad Software) was used for statistical analysis and graphical representation of the data. Normality of the data was assessed using Shapiro-Wilk test. Correlations were performed with Spearman correlation. For pairwise comparisons between two groups, paired t test, unpaired t test, or Wilcoxon test was used. For more multiple comparisons between more groups, one-way ANOVA with multiple comparisons through Tukey test and non-parametrical Kruskal-Wallis test with Dunn’s multiple comparisons were performed. For cell viability and infectivity assays, and tumor growth control curves involving several time points, two-way ANOVA with Tukey multiple comparisons test was used. Results were considered statistically significant at *p* < 0.05. Data shown on plots are presented as mean (SD). ∗∗∗∗*p* < 0.0001, ∗∗∗*p* < 0.001, ∗∗*p* < 0.01, ∗*p* < 0.05, ns = non-significant.

## Data availability

All data are available upon request.

## Acknowledgments

This study was supported by the Doctoral Programme of Clinical Research and 10.13039/100007797University of Helsinki, 10.13039/501100004012Jane and Aatos Erkko Foundation, HUCH Research Funds (VTR), 10.13039/501100010711Cancer Foundation Finland, 10.13039/501100006306Sigrid Jusélius Foundation, 10.13039/501100003127Finnish Red Cross Blood Service, TILT Biotherapeutics Oy. European Union’s Horizon 2020 research and innovation programme under the Marie Skłodowska-Curie grant agreement (no. 813453). We thank Albert Ehrnrooth and Karl Fazer for research support. We thank Minna Oksanen and Sini Raatikainen for expert experimental and administrative assistance. We also thank the Laboratory Animal Center (LAC, University of Helsinki, Helsinki, Finland) and the Biomedicum Flow Cytometry Unit (University of Helsinki, Helsinki, Finland). Open access was funded by the Helsinki University Library (10.13039/100007797University of Helsinki, Finland).

## Author contributions

V.A., T.V.K., J.H.A.C., D.C.A.Q., J.M.S., V.C.-C., and A.H. designed the experiments. V.A., T.V.K., J.H.A.C., S.B., S.A.P., E.J., L.H., M.V.d.H., and N.O. conducted the experiments. V.A., T.V.K., J.H.A.C., E.J., M.V.d.H., and L.H. collected the samples. V.A., T.V.K., J.H.A.C., S.A.P., E.J., M.V.d.H., S.B., and A.H. analyzed the results. All the authors contributed to writing and reviewing the manuscript.

## Declaration of interests

A.H. is a shareholder of Circio Holdings ASA (Norway). A.H., V.C.-C., J.H.A.C., J.M.S., D.C.A.Q., and L.H. are employees of TILT Biotherapeutics, Ltd (Finland). A.H., V.C.-C., J.C., J.M.S., and D.C.A.Q. are shareholders of TILT Biotherapeutics, Ltd (Finland).

## Supplemental information

Document S1. Figures S1–S4, and Tables S1–S3

Document S2. Article plus supplemental information
