# Supplementary material for: Boosting anti-tumor immunity with TILT-517 oncolytic adenovirus and checkpoint blockade in renal cell carcinoma
Source: Mol Ther Oncol. 2025 Apr 3;33(2):200979. doi: 10.1016/j.omton.2025.200979 (PMC12208612; doi:10.1016/j.omton.2025.200979)
Supplement: Document S1. Figures S1–S4, and Tables S1–S3 [file mmc1.pdf]

OMTON, Volume *33*

Supplemental information

Boosting anti-tumor immunity with TILT-517 oncolytic adenovirus and checkpoint blockade in renal cell carcinoma

Victor Arias, Tatiana V. Kudling, James H.A. Clubb, Elise Jirovec, Santeri A. Pakola, Mirte Van der Heijden, Saru Basnet, Dafne C.A. Quixabeira, Lyna Haybout, Nea Ojala, Susanna Grönberg-Vähä-Koskela, Anna Kanerva, Antti Rannikko, João M. Santos, Victor Cervera-Carrascon, Otto Hemminki, and Akseli Hemminki

Table S1: Patient Characteristics from each sample obtained. *ccRCC* – Clear Cell Renal Cell Carcinoma; *pRCC* – Papillary Renal Cell Carcinoma; IO – Immuno-oncology treatment; TKI – Tyrosine Kinase Inhibitor; SSIGN/5y CSS% - SSIGN Score/5-year estimated cancer-specific survival.

Sample – Patient ID	Age	Sex	Diagnosis	TNM	Grade	Necrosis	Stage	SSIGN/5y CSS% (Only ccRCC)	Prior cancer treatments
HR5	65	F	ccRCC	pT3aN0Mx	3	No	III	5 // 74.1% 5-year survival	No
HR6	46	M	ccRCC	pT2bN1M1	4	Yes	IV	14 // 19.2% 5-year survival	No
HR8	61	F	ccRCC	pT3bNxM0	2	Yes	III	6 // 74.1% 5-year survival	No
HR5	66	M	ccRCC	pT3aN0Mx	4	Yes	III	-	3line treatment (IO), stable with Sutent (sutinib, TKI)
HR16	74	M	ccRCC	pT3bN0Mx	3	Yes	III	7 // 38.6% 5-year survival	No
HR17	66	M	ccRCC	pT3bN0Mx	3	Yes	III	7 // 38.6% 5-year survival	No
HR18	66	M	ccRCC	pT4NxMx	4	Yes	IV	11 // 19.2% 5-year survival	No
HR19	42	F	pRCC	pT3bN1Mx	-	Yes	III	-	No
HR22	68	M	ccRCC	pT3aN0M0	2	No	III	4 // 89.8% 5-year survival	No

Table S2 – List of antibodies and panels used for experiments.

Baseline Panel 1 (All cell types)			
**Name**	**Fluorochrome**	**Clone**	**Brand**
CD45	Alexa Fluor 488	QA17A19	BioLegend
CD31	PE	WM59	BioLegend
CA IX	Brilliant Violet 711	M75	BD Biosciences
CD10	PE-Cy5	HI10a	BioLegend
EpCam (CD326)	Brilliant Violet 605	9C4	BioLegend
**Baseline Panel 2 (Immune cells)**			
**Name**	**Fluorochrome**	**Clone**	**Brand**
CD3	Alexa Fluor 700	HIT3a	BioLegend
CD4	Brilliant Violet 570	OKT4	BioLegend
CD8a	PE-Cy7	RPA-T8	BD Biosciences
CD45	Alexa Fluor 488	2D1	BioLegend
CD56	Brilliant Violet 711	HCD56	BioLegend
CD11c	Brilliant Violet 421	3.9	BioLegend
**Activation and Exhaustion**			
**Marker**	**Fluorochrome**	**Clone**	**Brand**
CD45	Alexa Fluor 488	QA17A19	BioLegend
CD3	Alexa Fluor 700	HIT3a	BioLegend
PD-1	APC-Cy7	EH12.2H7	BioLegend
CD4	Brilliant Violet 570	RPA-T4	BioLegend
CD56	BV711	HCD56	BioLegend
CD8a	PE-Cy7	SK1	BioLegend
**Memory**			
**Marker**	**Fluorochrome**	**Clone**	**Brand**
CD45	Alexa Fluor 488	QA17A19	BioLegend
CD3	Alexa Fluor 700	HIT3a	BioLegend
CD45RA	Brilliant Violet 650	HI100	BioLegend
CCR7	APC	2-L1-A	BD Biosciences
**Animal Experiment with Syrian Hamster – Tumor microenvironment study**			
**Marker**	**Fluorochrome**	**Clone**	**Brand**
CD4	PE-Cyanine7	GK1.5	ThermoFisher
CD8	PE	eBio341	ThermoFisher
MHC-II	FITC	14-4-4S	ThermoFisher
CD51	APC	P1F6	Biolegend

Table S3 – List of primers used for qPCR experiments.

n	Target gene and primer	Sequence
1	Hexon – Forward	5’ – CCT ACA CCA ACA CAA CAA CTC– 3’
2	Hexon – Reverse	5’ – ATC CAC CTC AAA AGT CAT GTC – 3’
3	Hexon – Probe	5’ – [FAM] AAA CCT TCT CTA CGC CAA CTC CGC CCA [TAM] – 3’
4	Human b-actin – Forward	5’ – TCA CCC ACA CTG TGC CCA TCT-3– 3’
5	Human b-actin – Reverse	5’ – GTG AGG ATC TTC ATG AGG TAG TCA GTC– 3’
6	Human b-actin – Probe	5’ – [FAM] ATG CCC TCC CCC ATG CCA TCC TGC GT [TAM] – 3’
7	E4 – Forward	5’ – GGA GTG CGC CGA GAC AAC– 3’
8	E4 – Reverse	5’ – ACT ACG TCC GGC GTT CCAT – 3’
9	E4 – Probe	5’ – [FAM] GGC ATG ACA CTA CGA CCA ACA CGA TCT [TAM] – 3’
10	Hamster g-actin – Forward	5’ – GGT GCT TCT GACC GAGG – 3’
11	Hamster g-actin – Reverse	5’ – GCC TGA ATG GCC ACG TACA– 3’
12	Hamster g-actin – Probe	5’ – [FAM] TGG GGT GTT GAA GGT TTC AAA CAT [BBQ] – 3’

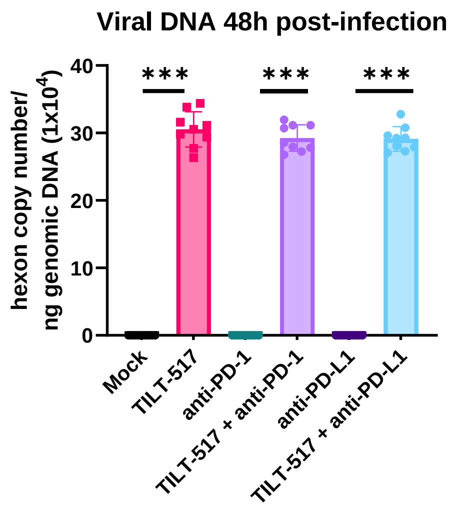

Figure S1: qPCR analysis from infected samples after 48h of infection. qPCR results for hexon viral gene from DNA extracted of the samples in each condition after 48h of infection. For statistical comparisons, non-parametrical Kruskal-Wallis with Dunn's multiple comparisons test was used. All data is presented as mean (SD). ***p<0.001.

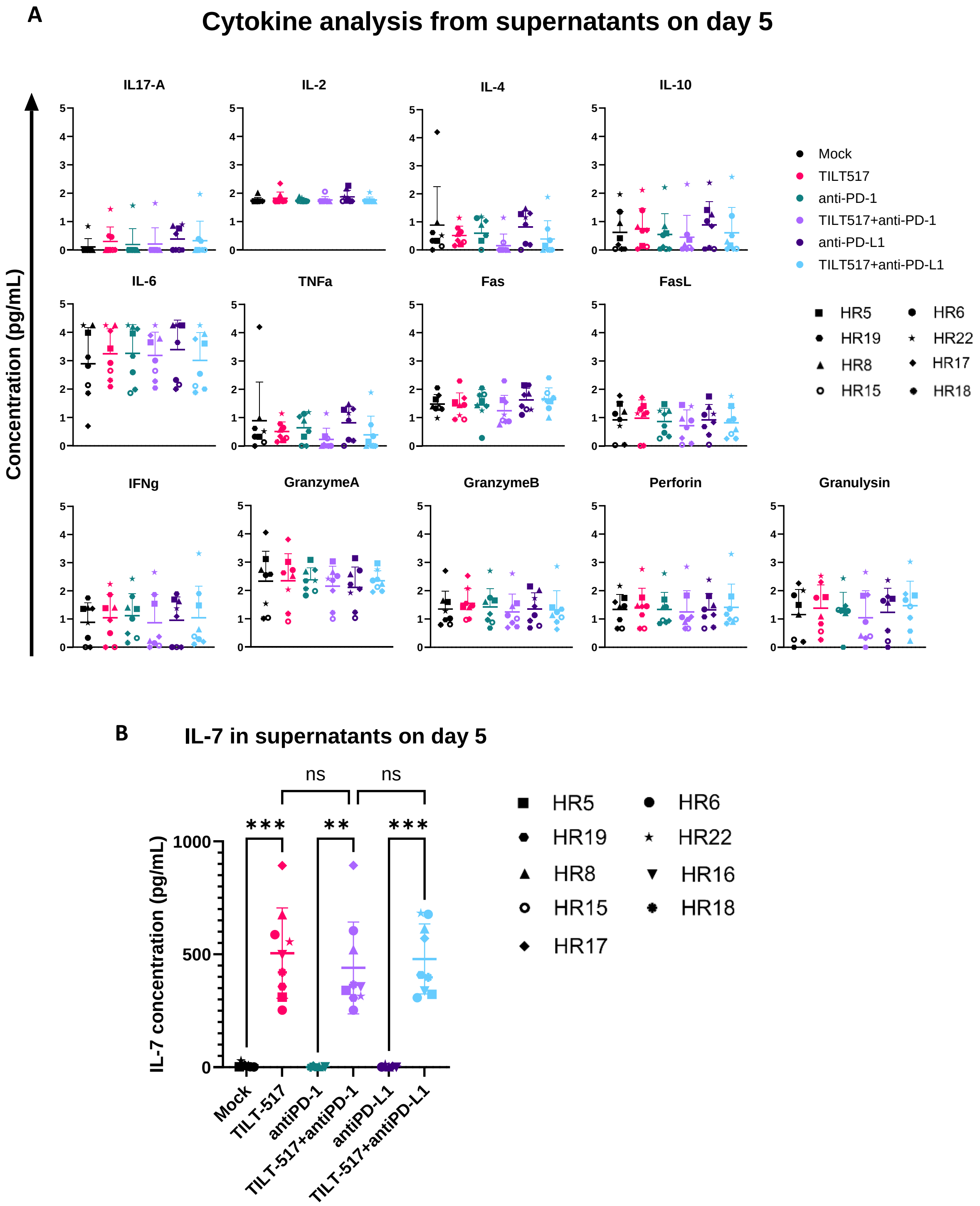

Figure S2: (A) Cytokine expression differences across RCC patient-derived samples after 5 days of treatment – Pairwise comparisons. Cytokine analysis results clustered by analyte and grouped by treatment condition. **(B) hIL-7 concentration in supernatants after 5 days of treatment**. RM one-way ANOVA with Tukey's multiple comparisons test was used to assess difference between treatment groups. All data is presented as mean (SD).***p<0.001,**p<0.01, ns=non-significant.

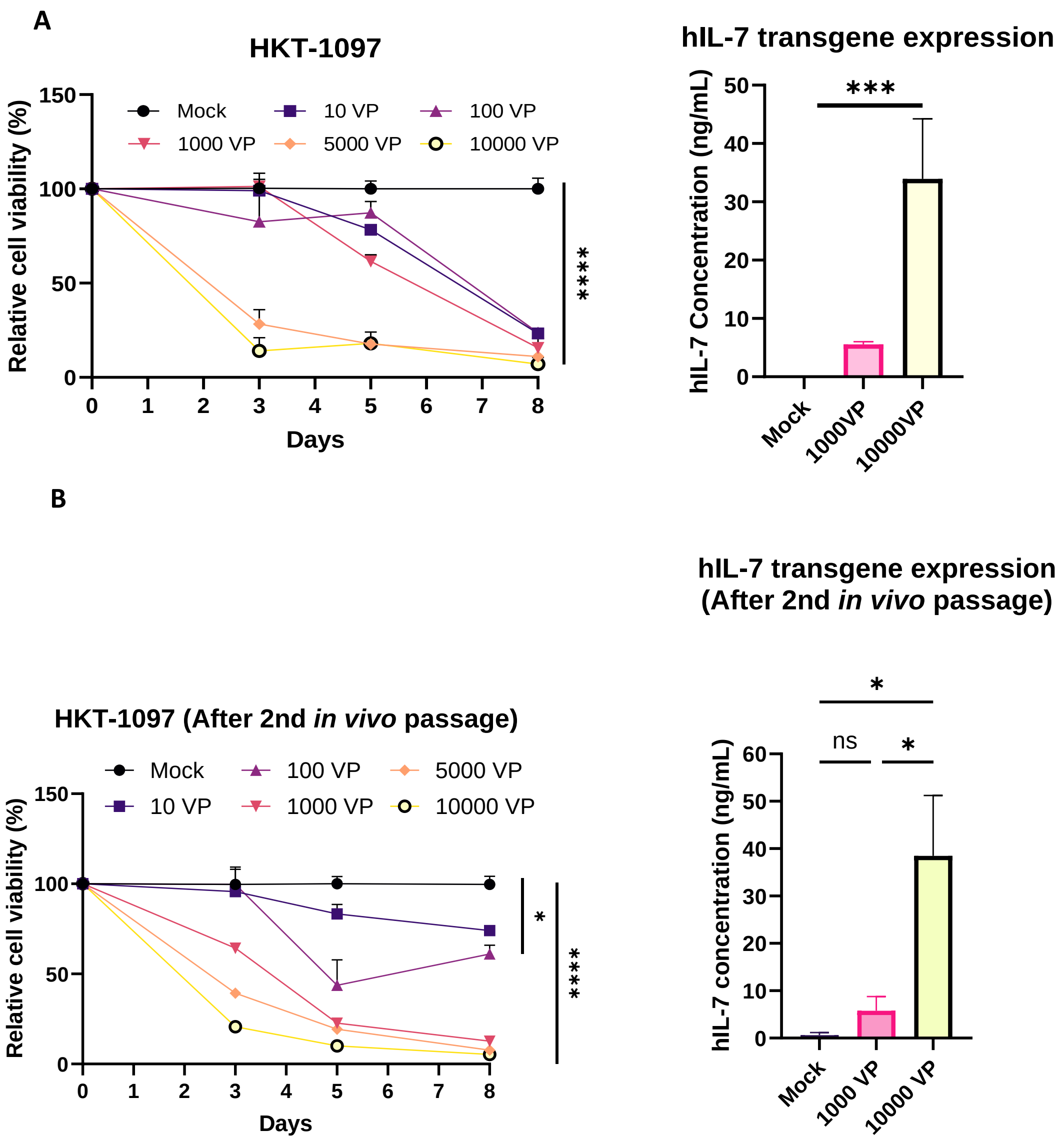

Figure S3: HKT-1097 in vitro studies with TILT-517. HKT-1097 cell line was selected for animal engraftment based on results on infectivity and expression of hIL-7 transgene in (A) the initial *in vitro* and in (B) the *in vivo* adapted cell line before engraftment. Infectivity of TILT-517 in HKT-1097 was measured in different VP/cell conditions (0VP or Untreated, 10VP, 100VP, 1000VP, 5000VP, 10000VP), and results were normalized to untreated. Expression of hIL-7 transgene was measured in HKT-1097 culture supernatant by day 3 on different VP/cell conditions (Untreated, 1000VP, 10000VP). Experiments were run in triplicates. For the infectivity assay, statistical significance was tested through 2-way ANOVA with Tukey multiple comparisons test, whereas for transgene expression, statistical significance was achieved by performing unpaired t-test. All data is presented as mean (SD). ****$p<0.0001$,***$p<0.001$,*$p<0.05$, ns=non-significant.

A

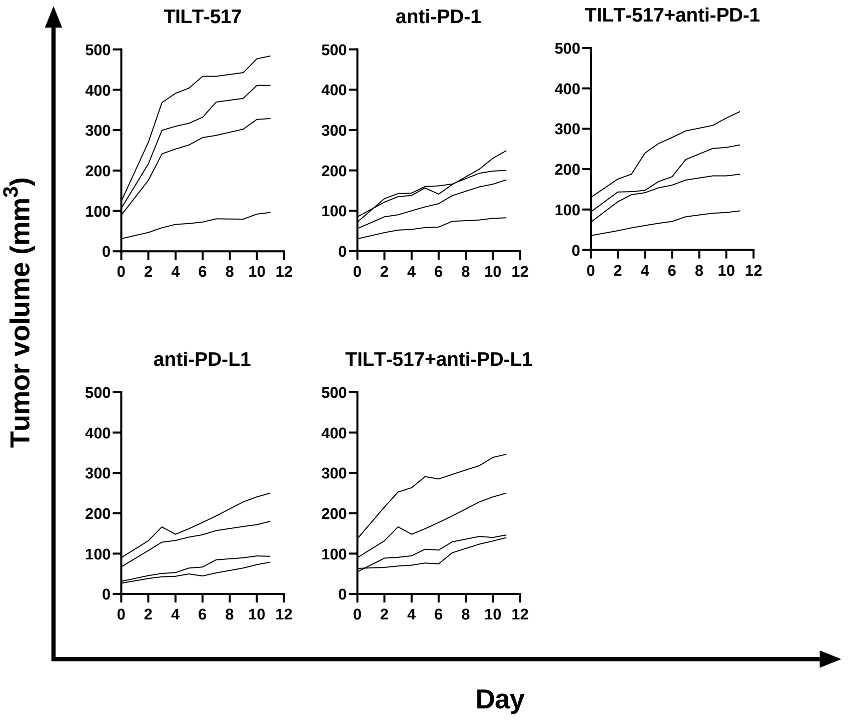

B

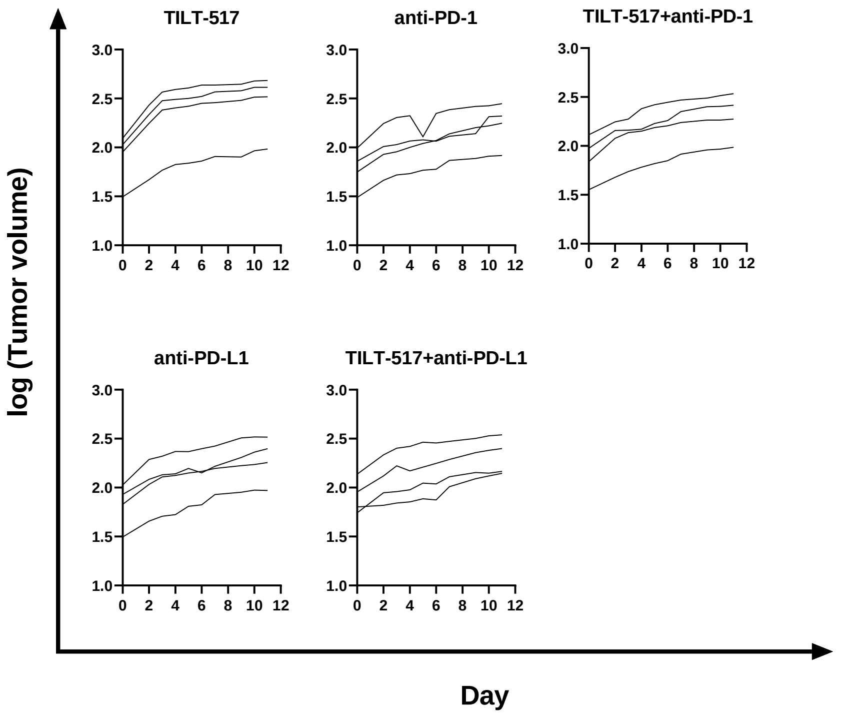

Figure S4: Unnormalized individual tumor growth curves per treatment condition: (A) Absolute individual tumor values, (B) log transformed individual tumor values. Tumors were measured in the span of 11 days, when the individuals were euthanized and the tumors collected.
